# Danggui Buxue Tang, a simple Chinese formula containing Astragali Radix and Angelicae Sinensis Radix, stimulates the expressions of neurotrophic factors in cultured SH-SY5Y cells

**DOI:** 10.1186/s13020-017-0144-y

**Published:** 2017-08-22

**Authors:** Amy G. W. Gong, Huai Y. Wang, Tina T. X. Dong, Karl W. K. Tsim, Y. Z. Zheng

**Affiliations:** 1 HKUST Shenzhen Research Institute, Hi-Tech Park, Nanshan, Shenzhen, 518000 China; 2 Division of Life Science and Center for Chinese Medicine, The Hong Kong University of Science and Technology, Clear Water Bay, Hong Kong, China; 3 Department of Biology, Hanshan Normal University, Chaozhou, 521041 Guangdong China

**Keywords:** Danggui Buxue Tang, Neuronal functions, SH-SY5Y cells

## Abstract

**Background:**

Danggui Buxue Tang (DBT), a phytoestrogen-enriched Chinese herbal formula, serves as dietary supplement in stimulating the “*Blood*” functions of menopausal women. In traditional Chinese medicine (TCM) theory, *“Blood”* has a strong relationship with brain activities. Previous studies supported that some ingredients of DBT possessed neuronal beneficial functions. Therefore, the neurotrophic function and the mechanistic action of DBT were systematically evaluated in cultured human neuroblastoma SH-SY5Y cells.

**Methods:**

The DBT-triggered protein expressions were analyzed by western blotting, while the transcriptional activities of promoters coding for related genes were revealed by luciferase assays. For mechanistic analysis of DBT, Erk1/2 and its inhibitor U0126 were analyzed.

**Results:**

The application of DBT in cultured neuroblastoma cells showed the efficacies in: (1) up-regulation of nerve growth factor (NGF), brain-derived neurotrophic factor (BDNF) and glial cell line-derived neurotrophic factor (GDNF); (2) activation of transcriptional activities of promoters coding for NGF, BDNF, GDNF; (3) activation of Erk1/2 and CREB; and (4) attenuation of the neurotrophic factor expression by the treatment of an Erk1/2 inhibitor.

**Conclusions:**

Our study supports that MAPK/Erk pathway acts as fundamental role in monitoring DBT-induced expression of neurotrophic factors in cultured human neuroblastoma cell. These results shed light in developing the working mechanism of this ancient herbal decoction for its neuronal function.

**Electronic supplementary material:**

The online version of this article (doi:10.1186/s13020-017-0144-y) contains supplementary material, which is available to authorized users.

## Background

Danggui Buxue Tang (DBT), a traditional Chinese medicine (TCM) herbal decoction, includes two common herbs, Astragali Radix (AR) and Angelicae Sinensis Radx (ASR) at the weight ratio of 5 parts to 1 part [1, 2]. DBT was recorded in ***Neiwaishang Bianhuo Lun*** by Li Dongyuan in AD 1247. Traditionally, DBT is utilized in enriching “*Blood*” and nourishing “*Qi*”. Today, DBT is suggested to be taken every day as a remedy for symptoms of menopause [3, 4]. Recent studies have revealed the pharmacological properties of DBT both in vivo and in vitro. In various animal models, the administration of DBT have shown effects in (1) enhancing population of red and white blood cells [5]; (2) stimulating estrogenic properties [6]; (3) increasing bone regeneration [7]; (4) triggering immune responses [8]; and (5) inducing formation of capillaries and blood vessels [9]. In parallel, those efficiencies were re-confirmed in cultured cells [3, 4].

AR, one of the most famous raw materials found in TCM herbal formulae, has abundantly amount of flavonoids, which exhibits similar functions to 17-β-estradiol [1, 2]. Formononetin, ononin, calycosin and calycosin-*7*-*O*-*β*-*d*-glucoside, the predominant bioactive components found within AR, possessed hematopoietic functions by stimulating protein expressions of hypoxia-inducible factor-1α (HIF-1α) and erythropoietin (EPO) in cultures [10]; the combination of these 4 flavonoids increased the hematological parameters in anemia rat models [11]. Besides, several lines of evidence supported the notion that flavonoids could have beneficial effects in human body on distinct aspects, including anti-tumor growth, anti-oxidation and neuronal beneficial functions [12]. Nevertheless, the possible role of flavonoid-enriched DBT decoction in neuronal function has not been illustrated.

Neurogenesis is a crucial turnover mechanism that rescues the number and survival of neurons. Neurogenesis involves neuronal regeneration, neuronal differentiation and synapse formation. The synthesis and secretion of neurotrophic factors, including nerve growth factor (NGF), brain-derived neurotrophic factor (BDNF) and glial cell line-derived neurotrophic factor (GDNF), are one of the major inducers for neurogenesis: these neurotrophic factors could regulate growth, survival and differentiation of neurons [13]. The MAPK/Erk transduction mechanism responding for external stimulations is activated under stressed condition. The inhibition of MAPK/Erk pathway was capable of stimulating oxidative stress and seizure-like activity in brain [14]. Furthermore, U0126, a MEPK/Erk specific inhibitor, was shown to protect primary cortical cultures against oxidative stress triggered by glutamate or hypoxia, suggesting that the activation of MAPK/Erk transduction plays a crucial role in regulating neuroprotection [15]. Here, we aimed at revealing the potential neuroprotection effects of DBT in cultures. A well-studied human neuroblastoma cell line, SH-SY5Y, was employed here to investigate the induction effect of DBT on neurotrophic factor expression as well as the signaling pathways being involved.

## Methods

### Raw materials and preparation of DBT formula

Three-year-old *Astragalus membranaceus* var. *mongholicus* (AR) and two-year-old *Angelica sinensis* roots (ASR) were collected. The raw materials were qualified according to analysis listed in China Pharmacopeia, and the microscope identifications were carried by Dr. Tina Dong. In order to produce DBT formula, the amounts of ASR and AR were preciously weighed at 1:5. The herbs were mixed well and then boiled twice in water [1, 2]. Before performing biological assay, the water extract was lyophilized and re-suspended in water at final concentration at 100 mg/mL. All the samples were kept at −80 °C.

### Fingerprint chromatograms of DBT

An Agilent 1200 series system (Agilent, Santa Clara, CA), supplied with auto-sampler, binary pump, degasser and thermo-stated column compartment, was involved here for chemical fingerprint analysis. Chromatographic conditions were performed on an Agilent, Eclipse Plus, C_18_ column (4.6 × 250 mm, 5 µm). Here, acetonitrile (as Solvent A) and 0. 1% formic acid (as Solvent B) were utilized as mobile phase, the flow rate was kept as 1.0 mL/min at room temperature. In brief, the chromatographic condition of DBT was shown here: 0–10 min, 15% of solvent A; 10–45 min, 15–50% solvent A; 45–50 min, 50% of solvent A; 50–70 min, 50–80% solvent A. All samples were able to pass through 0.45 µm Millipore syringe filter before injecting for analysis, and 10 µL was injected for HPLC. An ELSD detector and a DAD detector at an absorbance of 254 nm were used [2, 16].

### Cell culture

SH-SY5Y cells, a human neuroblastoma line, were purchased from American Type Culture Collection (ATCC, Manassas, VA, USA). In brief, cells were supplied with Dulbecco’s modified eagle’s medium (DMEM) with 10% fetal bovine serum (FBS), 100 units/mL penicillin and streptomycin in 37 °C incubator. DMEM, FBS and penicillin and streptomycin were obtained from Invitrogen Technologies (Carlsbad, CA, USA).

### Luciferase assay

Four promoter constructs were purchased from Addgene (Suite, MA), namely pBDNF-Luc, pGDNF-Luc, pNGF-Luc and pCRE-Luc carrying BDNF, GDNF, NGF, and CRE promoter sequences, respectively. Two hundred nanogram of each plasmid was transfected by Lipofectamine 3000 reagent (Invitrogen) in cultured SH-SY5Y cells. Cultured cells were seeded in 24-well plates at 6 × 10^4^ cell/mL, and then added various concentrations of drugs for 2 days. After drug treatment, the medium was aspirated, and PBS was utilized twice for washing cells. Luciferase lysis buffer was stored at 4 °C, containing 0.2% Triton X-100, 1 mM dithiothreitol (DTT) and 100 mM potassium phosphate, was employed here to lyse cell. Centrifugation at 13,200 rpm for 10 min, and then the supernatant was harvested and used to carry out luciferase assay (Tropix Inc., Bedford, MA, USA). Forskolin (FSK) served as a positive control.

### Western blot

The phosphorylations of Erk1/2 and CREB were analyzed here by western blot with using specific antibodies. Serum-starved were at least 3 h of cultured before drug applications. The cultures were collected immediately in 2× lysis buffer (125 mM Tris–HCl, 2% SDS, 10% glycerol, 200 mM 2-mercaptoethanol, pH 6.8) after drug/inhibitor (U0126, 10 µM)/activator (TPA, 100 nM) applications, and the samples were prepared for SDS-PAGE. After transferring, the membranes were incubated with 1: 5,000 dilutions of anti-phospho-Erk1/2 (Upstate, Lake Placid, NY, USA), 1:5000 dilutions of anti-phospho-CREB (Cell Signaling, Danvers, MA, USA) overnight and incubated at cold room. Before adding secondary antibody, TBST should be employed here for washing membranes 4 times, and each time at 10 min. Lastly, 1:5000 dilutions of horseradish peroxidase (HRP)-conjugated anti-rabbit secondary were incubated at 3 h at room temperature, the immune-complexes were observed by the enhanced chemiluminescence (ECL) method (Amersham Biosciences, Piscataway, NJ, USA). The band intensities were compared on an image analyze tool.

The expression levels of NGF, BDNF and GDNF were analyzed by western blot. In brief, cells were seeded onto 6-well plate, and after 2 days of drug/activators (FSK, 10 µM)/blockers (U0126, 10 µM) treatments, the cultures were washed by PBS twice and harvested in high salt lysis buffer (1 M NaCl, 10 mM HEPES, pH 7.5, 1 mM EDTA, 0.5% Triton X-100). After 10 min of centrifugation at 16,100 rpm, supernatant was kept for further step. Equal amount of sample protein was added by 2× lysis buffer and heated at 95 °C before subjecting to SDS-PAGE. The specific antibodies, i.e. anti-GDNF, BDNF and NGF antibodies (Cell Signaling) were incubated with membranes after transferring at 1:1000 dilutions at cold room for 12 h.

### Cell viability assay

MTT was employed for revealing cell viability. In brief, cells were seeded in 96-well plate. Drug treatments for 2 days, the final concentration of 0.5 mg/mL of MTT solution was applied into after 2 h durations, the production of purple crystal was dissolved in DMSO. The optimized absorbance was set at 570 nm.

### Statistical analysis and other assays

One-way analysis of variance was utilized for statistical tests. Data were expressed as Mean ± SEM, where *n* = 4–5. The highly significant was labeled as (***) where *p* < 0.001, more significant (**) where *p* < 0.01 and significant (*) where *p* < 0.05 compared with corresponding control group without U0126; (^^^) where *p* < 0.001, more significant (^^) where *p* < 0.01 compared with corresponding FSK or DBT group without U0126, respectively. The Minimum Standards of Reporting Checklist (Additional file 1) contains details of the experimental design, and statistics, and resources used in this study.

## Results

### Chemical standardization of DBT

Chemical standardization is the critical step for ensuring consistency and repeatability of biological experiments. DBT, prepared according to the optimized condition, was guaranteed the quality by chemical standardization [2, 16]. Chromatographic conditions of DBT were carried out by both ELSD and DAD detectors (Additional file 2), as reported previously [2, 16]. By quality control analysis, 1 g of dried DBT herbal extract, a qualified DBT extracts should consists of 809 µg of ASR-derived ferulic acid and 212 µg of Z-ligustilide, 693 µg of AR-generated calycosin and 164 µg of formononetin.

### DBT induces neurotrophic factor expressions

**Fig. 1:**
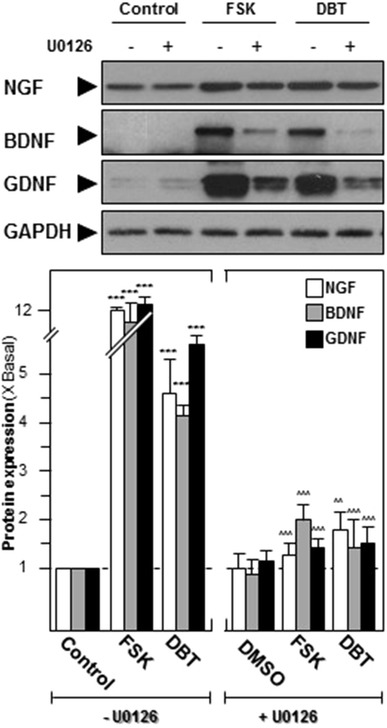
DBT induces the neurotrophic factor expressions in cultured SH-SY5Y cells. Cultured SH-SY5Y cells were pre-treated with fresh medium/U0126 (10 µM) for 3 h before application of this ancient herbal formula (0.5 mg/mL) for 2 days. The cell lysates were collected to determine the protein expressions by using specific antibody (*upper panel*). FSK (10 µM) acted as a positive control. Loading control was set as GAPDH. Protein expression level was calculated by a densitometer (*lower panel*). Values were expressed as the fold of increase as compared to untreated culture. Data were expressed as Mean ± SEM, where *n* = 4. **p < 0.01; ***p < 0.001 compared with corresponding control group without U0126. ^^*p* < 0.01; ^^^*p* < 0.001 compared with corresponding FSK or DBT group without U0126, respectively

The productions of neurotrophic factors, i.e. NGF, BDNF, GDNF, are essential for neuronal survival, growth and differentiation [17]. The deficiencies of neurotrophic factors could lead to malfunction of the nervous system, resulting in various kinds of neurological disorders. We tested both transcriptional activities and protein expressions of NGF, BDNF, and GDNF in cultured SH-SY5Y cells. Forskolin (FSK), an inducer of cAMP, was shown to induce neurite outgrowths and neurotrophic factor productions [18]. Application of 10 µM of FSK in cultures was employed as a positive control, having ~12- , ~10- and ~15-fold of increase in NGF, BDNF and GDNF protein expressions, respectively **(**Fig. 1). Before performing the bioassay, MTT assay was performed to ensure the maximal concentration of the herbal extract, and the results indicated that the highest concentration of DBT should be less than 1 mg/mL (Data not shown here). DBT extract (0.5 mg/mL) was added onto SH-SY5Y cultures for 2 days, and it could induce the expressions of neurotrophic factors, i.e. NGF at ~4.5-fold, BDNF at ~fourfold and GDNF at ~sixfold (Fig. 1). The activations of Erk1/2 and CREB were proposed to be the predominant mechanisms for production of neurotrophic factors [17]. In line to this notion, the pre-treatment of Erk1/2 inhibitor, U0126, could attenuate the activation of neurotrophic factors, induced by DBT herbal decoction (Fig. 1).

**Fig. 2:**
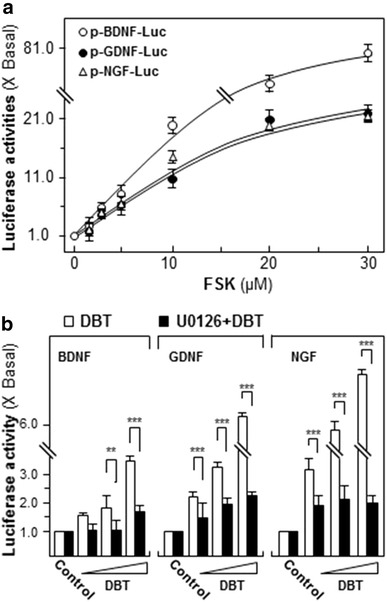
DBT stimulates transcriptional activities of NGF, BDNF and GDNF in cultured SH-SY5Y cells. Cultured cells, transfected with pBDNF-Luc or pGDNF-Luc or pNGF-Luc, were subsequently treated with **a** FSK (1–30 µM) or **b** DBT decoction (0.125, 0.25, 0.5 mg/mL) for 48 h. Cells were collected to determine the luciferase activity. The treatment of U0126 (10 µM) was 3 h before DBT application. Promoter-driven luciferase was expressed as the ratio to the negative control. All values were revealed as Mean ± SEM, where *n* = 5. **p < 0.01; ***p < 0.001 compared with control

The transcriptional activities of NGF, BDNF and GDNF were also revealed here. The promoter constructs of neurotrophic factors tagged with luciferase, i.e. pNGF-Luc, pBDNG-Luc and pGDNF-Luc, were employed here. In the transfected SH-SY5Y cells, FSK stimulated the transcriptional activities in a concentration-dependent manner. The highest induction was reached at 20 µM, i.e. ~80-fold for BDNF, ~20-fold for GDNF and ~20-fold for NGF (Fig. 2a). In parallel, the application of DBT induced the transcriptional activities of NGF, BDNF and GDNF in a dose-dependent manner; the maximal stimulations were revealed at ~eightfold for NGF, ~3.5-fold for BDNF, ~sixfold for GDNF (Fig. 2b). Again, the pre-treatment of U0126 suppressed markedly the DBT-induced transcriptional activation of neurotropic factor (Fig. 2b).

### DBT induces Erk1/2 and CREB phosphorylation

**Fig. 3:**
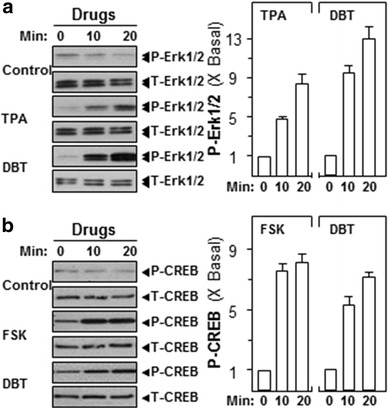
DBT induces the phosphorylation of Erk1/2 and CREB. Cultured SH-SY5Y cells were serum-starved for 3 h before the application of DBT (0.5 mg/mL) for different duration. **a** Total Erk1/2 (T-Erk1/2) and phosphorylated Erk1/2 (P-Erk1/2) and **b** total CREB (T-CREB) and phosphorylated CREB (P-CREB) were analyzed by specific antibodies (*left panel*). TPA (100 nM) and FSK (10 µM) played as positive control for the activation of Erk1/2 and CREB, respectively. The band density was measured by densitometer (*right panel*), and the phosphorylation values were expressed as the ratio to the basal reading where the time zero equaled to 1, values were expressed as Mean ± SEM, where *n* = 4

**Fig. 4:**
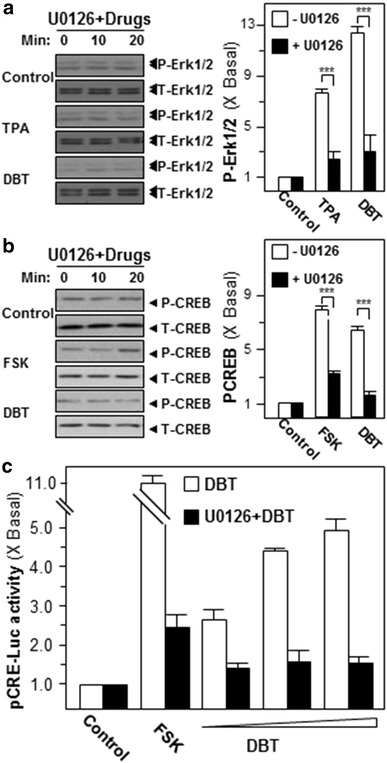
U0126 attenuates the activations of Erk1/2, CREB and CRE. Cultured SH-SY5Y cells were pre-treated with 10 µM U0126 for 3 h before the herbal treatment (0.5 mg/mL) for different duration. **a** Total Erk1/2 (T-Erk1/2) and phosphorylated Erk1/2 (P-Erk1/2) and **b** total CREB (T-CREB) and phosphorylated CREB (P-CREB) were investigated by specific antibodies (*left panel*). TPA (100 nM) and FSK (10 µM) served as positive control for activation of Erk1/2 and CREB, respectively. The band density was measured by densitometer (*right panel*). **c** In pCRE-Luc transfected cultures, cells were pre-treated with fresh medium/U0126 (10 µM) for 3 h before the treatment of DBT (0.125, 0.25, 0.5 mg/mL) for 48 h. Cells were collected to determine the luciferase activity. FSK (10 µM) served as a positive control. **p < 0.01; ***p < 0.001 compared with control

The stimulations of Erk1/2 and CREB are the predominant mechanism for neurotrophic factor production [17]. In cultured SH-SY5Y cells, application of DBT induced Erk1/2 phosphorylation in a time-dependent manner; the maximal activation was revealed ~13 folds at ~20 min (Fig. 3a). Furthermore, we investigated the downstream signaling of Erk1/2, i.e. the inducer of CREB phosphorylation. Similar to our analysis of Erk1/2 phosphorylation, the treatment of DBT induced CREB phosphorylation in a time-dependent manner: the maximal induction was revealed at 20 min of ~sixfolds of activation (Fig. 3b). TPA, the activator of Erk1/2, and FSK served as a positive control for phosphorylations of Erk1/2 and CREB, respectively (Fig. 3). Here, U0126 was utilized to further specify the cell signaling pathways. In the presence of U0126 (10 µM), the phosphorylations of Erk1/2 and CREB were significantly suppressed in DBT-treated cells (Fig. 4a, b**)**. Moreover, we also tested the transcriptional activity of cyclic AMP responsive element (CRE), a key factor to promote neurogenesis. DBT induced the promoter activation of pCRE-Luc-transfected cells in a concentration-dependent manner with a maximum of ~fivefolds activation at 0.5 mg/mL (Fig. 4c). Nevertheless, the pre-treatment of U0126 significantly attenuated the DBT-stimulated pCRE-Luc transcriptional activities as shown in Fig. 4c.

## Discussion

According to TCM theory, the herbal combination was believed to enhance the therapeutic efficacy of single herb via herbal compatibility [19]. Synergistic works among different herbs had been well illustrated in DBT [4]. For example, the boiling AR and ASR together could generate a perfect decoction having the best chemical and biological properties [1, 10]. Traditionally, DBT is prescribed to improve menopausal symptoms [2]. In addition, the efficacies of DBT have been revealed and confirmed in different aspects, i.e. estrogenic effect, bone development, blood enhancement, immune stimulation and cardiovascular function [1, 2, 16]. Here, we proposed a possible function of DBT in the brain.

The neuro-functions of AR and ASR, two herbs making up DBT, had been verified in enhancing memory and in promoting synaptic plasticity [20]. In particular, the flavonoids derived from AR, such as calycosin and formononetin, are regarded as an important and effective constituent within DBT. These flavonoids were shown to be absorbable by cells [10]. Several types of flavonoids are believed to show beneficial effects on neural stem cell for its differentiation and survive. Hesperidin was able to increase survival rate of neural crest cells [20]. Pre-treatment of quercetin, a flavonol, was capable to prevent H_2_O_2_-induced cellular viability [21]. Furthermore, Baicalein, a flavone, was shown to protect neural progenitor cells from irradiation-induced necrotic cell apoptosis by elevating the BDNF-mediated signaling in hippocampus [22]. It also reported that flavonoids, and their known physiologically relevant metabolites, were able to cross the blood–brain barrier using well-established in vitro models, i.e. brain endothelial cell lines and ECV304 monolayers co-cultured with C6 glioma cells [23]. The intake of isoflavonoid-enriched herbal decoction in rat could induce productions of neurotrophic factors, and subsequently the decoction rescued cognitive impairment associated with N-methyl-D-aspartate (NMDA) receptor antagonism [24], promoted hippocampal neurogenesis [25] and attenuated depressive symptoms [26]. Aging and Alzheimer’s disease (AD) are characterized by deficiency of learning and memory. The close relationship between AD and aging plays a critical role in elucidating the pathophysiological mechanism in each event, e.g. the involvement of neurotrophic factors in both processes [27]. BDNF is important in neuronal growth and neuronal survival, in particular the effect in synaptic processes of memory. Indeed, a decreased level of pro-BDNF was shown in mild cognitive impairment (MCI) patients [25]. The intake of NGF in AD patients showed improvements in cognitive functions, as well as a low level of amyloid β in cerebrospinal fluid [28]. Moreover, a reduced level of GDNF led to excess glutamate release and deregulation of glutamate transporter-1, which caused the excitotoxicity in nervous system [29].

Neurons are responsible to process and transmit information within human body. MAPK/Erk pathway is a key factor of NMDA receptor signaling transduction in regulating neuronal development, synaptic communications and neuroplasticity. The stimulated MAPK/Erk mechanism is believed to contribute AD pathogenesis via multiple mechanisms, e.g. up-regulation of neuronal apoptosis, transcriptional and translational activations of β- and γ-secretases, and stabilization and phosphorylation of amyloid precursor protein [30]. In fact, the initiation of MAPK/Erk signaling can trigger the activation of cAMP response element binding protein (CREB) [31]. CREB activation is important in gene transcriptions, in particular during the promotion of cell survival [32]. Here, the application of this ancient herbal formula was capable of inducing the activations of Erk and CREB in a time-dependent manner. More importantly, the activations of Erk1/2 and CREB could be blocked by U0126. Thus, we believed that MAPK/Erk might involve in regulating neurotrophic expressions.

## Conclusion

We revealed that DBT could up regulate the expressions of neurotrophic factors via MAPK/Erk signaling mechanism. Furthermore, we believed that MAPK/Erk signaling pathway could act as the fundamental role in regulating neuronal functions. Therefore, DBT shed light as health supplements or therapeutic treatments for neurodegenerative diseases, e.g. possible treatment of AD.

## Additional files

**Additional file 1.** The Minimum Standards of Reporting Checklist.

**Additional file 2.** Ten µL of 100 mg/mL of herbal extracts were subjected to HPLC analysis, and the chromatographic were revealed at 254 nm by a UV detector and an ELSD detector. The typical fingerprint of DBT decoction was shown here.

## Abbreviations

AD: Alzheimer’s disease
AR: Astragali Radix
ASR: Angelicae Sinensis Radix
ATCC: American Type Culture Collection
BDNF: brain-derived neurotrophic factor
CRE: cyclic AMP responsive element
DBT: Danggui Buxue Tang
DMEM: Dulbecco’s modified Eagle’s medium
DTT: dithiothreitol
ECL: enhanced chemiluminescence
FSK: forskolin
GDNF: glial cell line-derived neurotrophic factor
HRP: horseradish peroxidase
MCI: mild cognitive impairment
NGF: nerve growth factor
NMDA: N-methyl-D-aspartate
TCM: traditional Chinese medicine
