# Supplementary figures and images for: Danggui Buxue Tang, a simple Chinese formula containing Astragali Radix and Angelicae Sinensis Radix, stimulates the expressions of neurotrophic factors in cultured SH-SY5Y cells
Source: Chin Med. 2017 Aug 22;12:24. doi: 10.1186/s13020-017-0144-y (PMC5568261; doi:10.1186/s13020-017-0144-y)

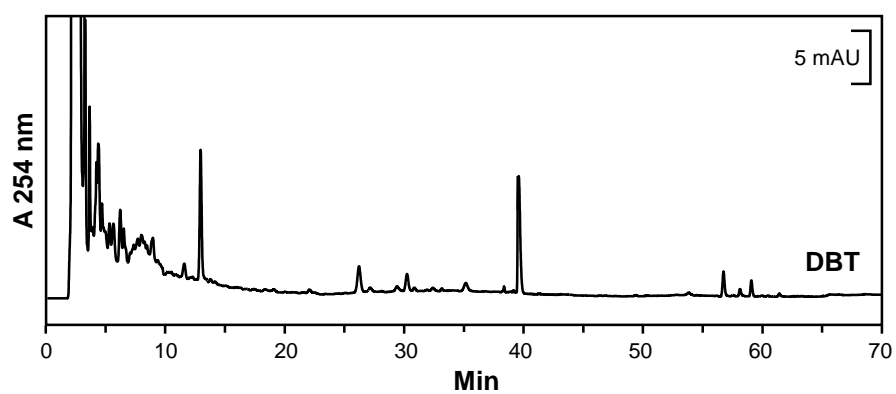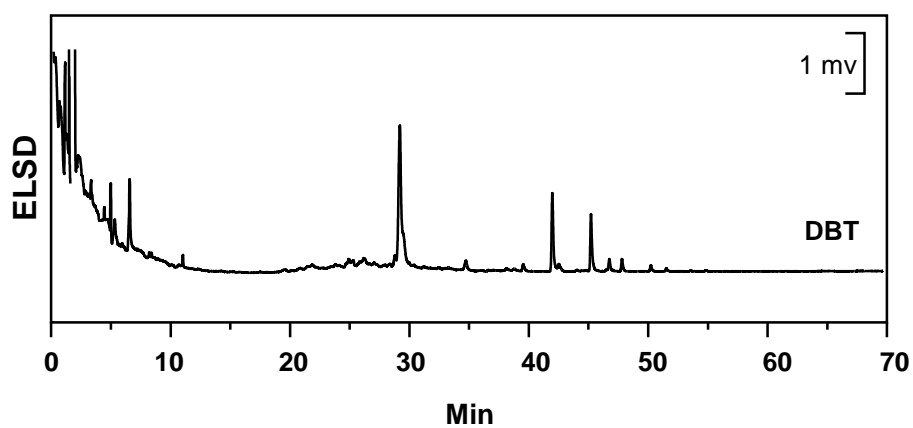

Supplement: Supplementary file 2 — Additional file 2. Ten µL of 100 mg/mL of herbal extracts were subjected to HPLC analysis, and the chromatographic were revealed at 254 nm by a UV detector and an ELSD detector. The typical fingerprint of DBT decoction was shown here. [file 13020_2017_144_MOESM2_ESM.pdf]
